# Nurse delivered lifestyle interventions in primary health care to treat chronic disease risk factors associated with obesity: a systematic review

**DOI:** 10.1111/j.1467-789X.2012.01029.x

**Published:** 2012-12

**Authors:** G M Sargent, L E Forrest, R M Parker

**Affiliations:** The Australian Primary Health Care Research Institute (APHCRI), The Australian National University (ANU), Australian Capital TerritoryCanberra, Australia

**Keywords:** Chronic disease prevention, lifestyle intervention, nursing, primary health care

## Abstract

Nurses in primary health care (PHC) provide an increasing proportion of chronic disease management and preventive lifestyle advice. The databases MEDLINE, CINAHL, EMBASE and PsychINFO were searched and the articles were systematically reviewed for articles describing controlled adult lifestyle intervention studies delivered by a PHC nurse, in a PHC setting. Thirty-one articles describing 28 studies were analysed by comparison group which revealed: (i) no difference of effect when the same intervention was delivered by a PHC nurse compared to other health professionals in PHC (*n* = 2); (ii) the provision of counselling delivered by a PHC nurse was more effective than health screening (*n* = 10); (iii) counselling based on behaviour change theory was more effective than the same dose of non-behavioural counselling when at least three counselling sessions were delivered (*n* = 3). The evidence supports the effectiveness of lifestyle interventions delivered by nurses in PHC to affect positive changes on outcomes associated with the prevention of chronic disease including: weight, blood pressure, cholesterol, dietary and physical activity behaviours, patient satisfaction, readiness for change and quality of life. The strength of recommendations is limited by the small number of studies within each comparison group and the high risk of bias of the majority of studies.

## Introduction

The international rise in obesity rates over the last three decades has been accompanied by an increase in preventable chronic diseases, such as type 2 diabetes, cardiovascular disease, stroke, arthritis and some cancers (1). Internationally, chronic diseases are managed in a variety of health care settings and their prevention is increasingly becoming a priority for primary health care (PHC) which is the first point of contact with the health system.

Nurses are an integral part of any multidisciplinary PHC team and have roles that continue to develop and expand in response to financial incentive, medical practitioner shortages and an imperative to decrease pressure on hospitals (2–5). Nurses in PHC are assuming an increasing proportion of the chronic disease management and preventive health advice (6). A systematic review of the literature of PHC nursing interventions provides strong international evidence to support the effectiveness of PHC nurses in a diverse range of roles including chronic disease management, illness prevention, health promotion and achievement of good patient compliance in treating chronic conditions, when assessed using quality of care measures (mortality, quality of care, compliance, knowledge, satisfaction), and use of resources (7).

Lifestyle change interventions focus on increasing healthy behaviours at the individual level and reducing chronic disease risk by controlling physiological variables known to be associated with chronic disease onset. Systematic reviews provide strong evidence that lifestyle interventions are effective in: preventing weight gain in adults who are obese (8), decreasing hypertension (8,9), positively affecting lipid levels (9), and reducing the onset of type 2 diabetes and the metabolic syndrome (8).

Little is known to inform the components of PHC nursing interventions for the prevention and management of chronic diseases associated with obesity. This is the first systematic review to compile the evidence regarding lifestyle change intervention effect, when delivered by PHC nurses, without restricting outcomes to those of cardiovascular disease risk (10). The aims of this research were to: (i) review the evidence of intervention effectiveness to change lifestyle risk factors when delivered by PHC nurses in a PHC setting; and (ii) inform the direction of future research to evaluate PHC nursing interventions to reduce lifestyle risk factors associated with overweight, obesity and preventable chronic diseases in adults.

## Methods

This systematic review was conducted and reported in accordance with Preferred Reporting Items for Systematic Reviews and Meta-Analyses guidelines (11,12).

### Key question

The key question informing this systematic review was: What does the published literature report on the effectiveness of interventions for adults which aim to affect change in lifestyle risk factors for chronic diseases that are associated with overweight and obesity, when these are delivered by a PHC nurse in a PHC setting?

### Eligibility criteria

Articles were eligible if they: described interventions with a lifestyle change component, were delivered to adults by a PHC nurse in a PHC setting, and reported quantitative outcomes on risk factors associated with obesity including: anthropometric, physiologic, behavioural or psychosocial. Randomized and non-randomized controlled trials (RCTs and non-RCTs) were included.

Papers were limited to primary sources, published in English. Articles were excluded if they: did not report outcomes for adults; involved treatment of severe mental health disorders; involved pharmaceutical treatment or if participants were using medications that were likely to affect primary outcomes (e.g. anti-hypertensive when blood pressure was a primary outcome); or involved surgical treatment. Studies reporting effect on smoking cessation or alcohol intake were excluded where this was the main focus of intervention, and included, if there were lifestyle change outcomes of interest, as interventions focused on smoking cessation have been reported elsewhere (13). Articles that did not clearly describe the involvement of either a PHC setting or PHC nurse delivery were excluded. No restrictions were placed on the primary outcome measure, the year of publication, length of intervention, follow-up period or format of the comparison group. To the best of our knowledge, all articles were peer reviewed.

### Information sources

Major health and medicine databases of published literature, MEDLINE, CINAHL, PsychINFO and EMBASE, were searched in September 2010. The bibliographies of included articles were hand searched to locate articles not catalogued in these databases.

### Search strategy

The database search strategy (Supporting Information Table S1) was constructed with the assistance of a specialist librarian, using medical subject headings (MeSH), and five groups of keywords. Articles retrieved by the search strategy had at least one term from each of the five groupings: (i) PHC setting (including general practice, family practice, primary care, medical staff, nursing staff, physician's office or community health); (ii) nurse delivery (including nurse practitioners, practice nurses, occupational health nurses and public health nurses, community nurses or health visitors); (iii) intervention evaluation studies (including treatment, therapy, intervention, management assessment or delivery); (iv) lifestyle change interventions (including dietary, physical activity, behaviour, health education or chronic disease management); and (v) evaluation of outcomes associated with obesity treatment (anthropometric or behavioural). A sixth group limited results by excluding articles outside the scope of the review. Word truncation and wildcards allowed for variations in spelling and word endings. Database limits for English full text were applied. Search terms were adjusted slightly for each database.

### Study selection

A reference management program (EndNote X1.0.1, Thomson™) was used to manage the included articles and remove book chapters and theses. The search function was used to exclude articles when the title contained the following keywords that were outside the scope of this review: dialysis, urinary, eating disorder, HIV, oncology, haemodialysis, ulcer, literature review or guideline.

Using an inclusion/exclusion criteria checklist, two reviewers (LF and GS) independently screened the title and abstracts of articles resulting in 87% reviewer concordance. Non-concordant articles were resolved by consensus or retained for full-text review if agreement was not reached. Full-text articles were reviewed (GS and LF) using an eligibility checklist. If clarification was necessary, the article was independently reviewed by a second reviewer (RP, LF or GS). Further library searches were conducted using the names of authors of included studies to identify subsequent or preliminary papers for those studies. The paper reporting the post-intervention outcome measures was regarded as the primary source.

### Data extraction

Data from publication describing included studies were extracted systematically by one reviewer (GS) into a database described elsewhere (14,15). No further information was sought from the authors. Data describing interventions that were reported in more than one article were extracted together. A second reviewer (LF) verified outcome tables.

### Data items

The following components of each intervention were recorded for comparative purposes: study design, intervention setting, setting recruitment, the involvement of PHC nurse/s in intervention delivery, personnel training as part of the intervention, behaviour change targets, target participants, participant recruitment, group treatment (comparison and intervention groups), number of contacts, treatment duration and outcomes.

### Risk of bias in individual studies

The risk of bias was assessed for individual studies according to adequate control of: selection bias or allocation bias, detection bias, attrition bias and reporting bias (15,16). Each study was scored for methodological limitations and risks of bias during data extraction (Table 1 and Supporting Information Table S2). An overall indication of quality according to the methodological limitations and risk of bias is also indicated. Randomized and non-randomized trials were assessed using the same criteria and studies were not excluded on the basis of risk of bias.

**Table 1:** Summary of methodological limitations and risk of bias (full information is available as Supporting Information online)

First author and year	Validated measures adequacy	Randomization	Risk of selection or allocation bias	Blinding adequacy	Risk of performance and detection bias	Risk of attrition bias	Evidence of outcome measure reporting bias	Overall rating
Balch 1976 (21)	Unclear	Adequate	×	Not done	×	××	Nil	××
Baron 1990 (28)	Adequate	Adequate	××	Inadequate	×	✓	Yes	×
Gemson 1990 (38)	Unclear	Adequate	×	Unclear	×	×	Yes	××
Beresford 1992 (30)	Adequate	Adequate	××	Not done	×	×	Yes	××
Karvetti 1992 (26)	Adequate	Adequate	××	Not done	×	××	Nil	××
Robertson 1992 (42)	Adequate	Adequate	×	Adequate	✓	×	Nil	×
Neil 1995 (17)	Adequate	Adequate	✓	Adequate	✓	✓	Yes	✓
Sander 1996 (45)	Unclear	Adequate	××	Inadequate	×	××	Nil	××
Bakx 1997 (27)	Adequate	Adequate	××	Not done	×	××	Nil	××
Roderick 1997 (35)	Adequate	Adequate	××	Unclear	×		Yes	××
Anderson 1999 (39)	Adequate	Adequate	××	Not done	×	××	Nil	××
Naylor 1999 (34)	Adequate	Adequate	××	Not done		××	Nil	××
Sims 1999 (36)	Adequate	Not done	Non-RCT ××	Adequate	✓	×	Nil	××
Steptoe 1999 (33)	Unclear	Adequate	✓	Not done	×	×	Yes	××
Gold 2000 (24)	Adequate	Not done	Non-RCT ××	Not done	×	××	Nil	××
Dubbert 2002 (41)	Adequate	Adequate	×	Adequate	✓	✓	Nil	×
Ammerman 2003 (44)	Adequate	Adequate	×	Not done	×	×	Yes	××
Aittasalo 2004 (23)	Adequate	Adequate	××	Not done	×	×	Nil	××
Little 2004 (22)	Adequate	Adequate	✓	Inadequate	×	✓	Yes	×
Little 2004 (43)	Adequate	Adequate	✓	Not done	×	✓	Nil	×
Purath 2004 & 2005 (37,75)	Adequate	Adequate	×	Not done	×	×	Yes	××
Kinnunen 2007 (32)	Adequate	Not done	Non-RCT ×	Not done	×	✓	Nil	××
Kinnunen 2007 (31)	Adequate	Not done	Non-RCT ××	Not done	×	×	Nil	××
Speck 2007 (29)t	Adequate	Not done	Non-RCT ×	Not done	×	×	Yes	××
Lawton 2009 & Rose 2007 (18,19)	Adequate	Adequate	✓	Adequate	✓	✓	Yes	✓
McTigue 2009 (25)	Adequate	Not done	Non-RCT ××	Not done	×	×	Yes	××
Whittemore 2009 (20)	Adequate	Adequate	✓	Adequate	✓	✓	Nil	✓
Faucher 2010 (40)	Unclear	Adequate	×	Inadequate	×	××	Nil	××

✓ No serious limitations and low risk of bias; × Serious limitations and some risk of bias; ×× Very serious limitations and high risk of bias.

RCT, randomized controlled trial.

### Synthesis of results

Comparison groups were often recorded within group changes; however, the outcomes reported here are restricted to outcome measures that were significantly (*P* < 0.05) different from the comparison group. Outcomes reporting smoking cessation and change in alcohol consumption were not extracted.

Because of the heterogeneity of outcome measures, neither a meta-analysis nor evidence profile on outcomes was appropriate. Results are instead synthesized, presented and discussed according to comparison group. The methodological limitations and risk of bias are presented for each study in the outcome tables and are discussed descriptively.

## Results

**Figure 1:**
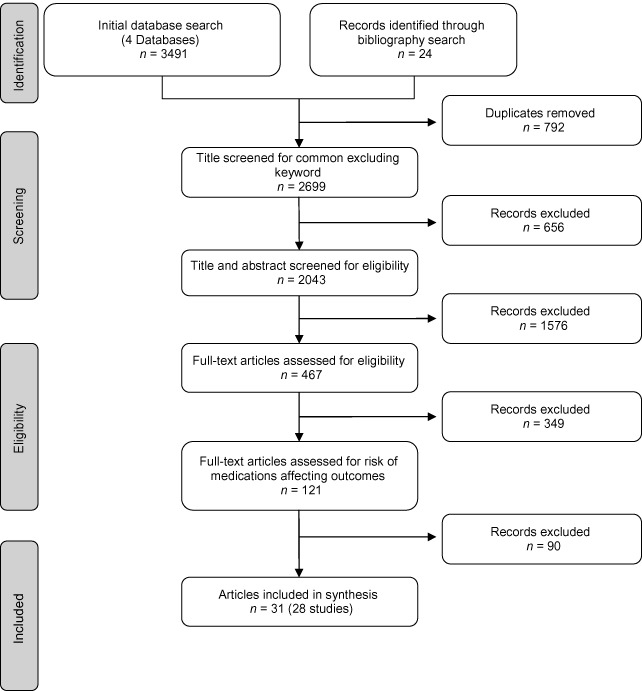
Flow of papers through selection process using Preferred Reporting Items for Systematic Reviews and Meta-Analyses format (11).

The database search identified 3,491 papers. The review process identified 31 articles describing 28 studies that were eligible for inclusion (Fig. 1). These studies involved a total of 10,759 participants and took place in the United Kingdom (*n* = 9), United States (*n* = 13), Finland (*n* = 4), the Netherlands (*n* = 1) and New Zealand (*n* = 1).

Twenty-two of the studies were RCTs, and the remaining six were non-RCTs. Three studies reported strong methodological rigour with no serious limitations and a low risk of bias (17–20). The remainder was assessed to have serious limitations and at least moderate (*n* = 5) or high (*n* = 20) risk of bias (Table 1).

About 14 of the 28 studies described nurses delivering behavioural counselling in an appointment between 5 and 30 min using theoretically based behaviour change techniques such as stage matching, motivational interviewing to enhance readiness for change or goal setting. Most of these described providing training prior to intervention delivery.

### Intervention delivery by primary health care nurses compared with other health professionals in primary health care

One study with no serious limitations and a low risk of bias (17) and one study with serious limitations and a high risk of bias (21) directly compared delivery of the same intervention by different health professionals in a PHC setting (Table 2). The interventions involved either two (17) or nine (21) contacts with a health professional. Significant changes were seen within all six treatment groups for anthropometric outcome measures over the short term with no adverse effects reported. There was no evidence that delivery by a PHC nurse, following brief training, affected outcomes differently compared to delivery by a dietitian (17), a psychologist or a social worker (21) (each with prior experience in delivering weight reduction counselling).

**Table 2:** Delivery by PHC nurses compared with other health professionals in PHC: intervention description, participant characteristics and outcomes of studies that compared effects. Results presented according to risk of bias, with lowest risk of bias first

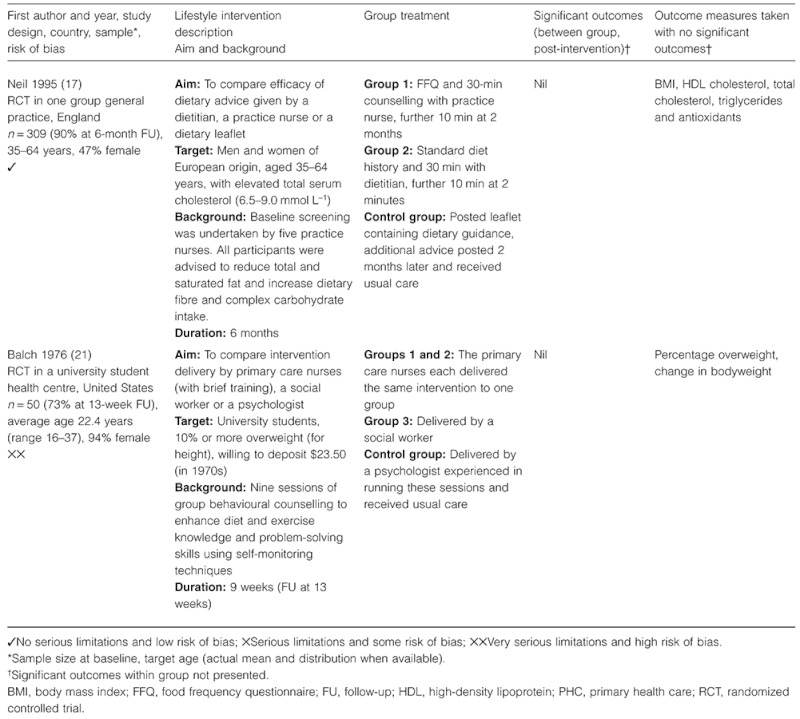

### Primary health care nurse counselling for lifestyle change compared with screening

There is good evidence (Table 3) from one high-quality study with a low risk of bias that behavioural counselling delivered by a nurse is significantly more effective than screening alone to increase physical activity levels and improve quality of life over a 1-year intervention, and that these may be maintained at a 2-year follow-up (18,19). This study did however observe more falls and injuries in the group of participants that undertook more physical activity and did not record significant anthropometric or physiological outcomes. One study with moderate risk of bias (22) indicated that 1 month of behavioural counselling may significantly affect positive changes in readiness and intent for physical activity when compared with screening alone. Two further studies, with a high risk of bias, offer supporting evidence that behavioural counselling is more effective than screening alone across a variety of outcomes (23,24).

**Table 3:** PHC nurse delivered behavioural counselling for lifestyle change compared with screening: intervention description, participant characteristics and outcomes of studies that compared effects. Results presented according to risk of bias, with lowest risk of bias first

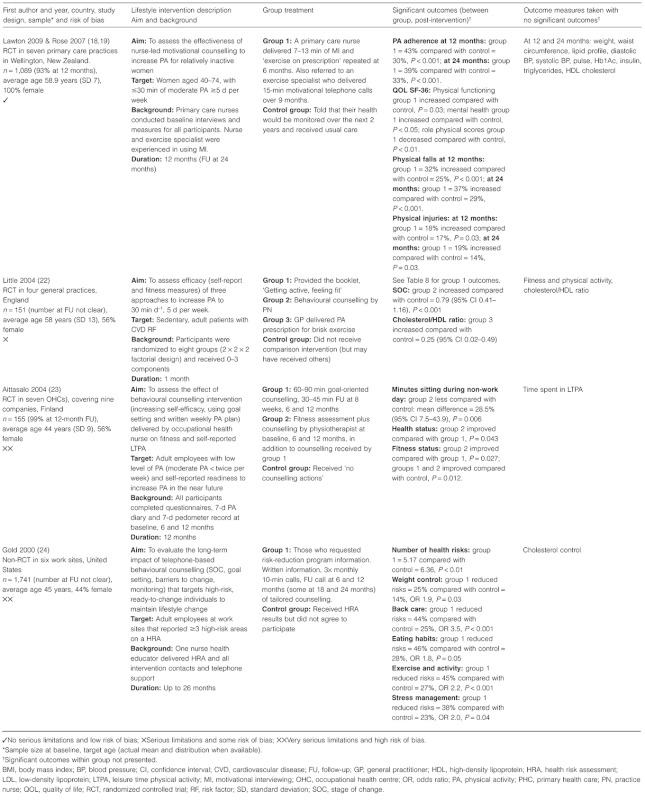

Six studies with a moderate (*n* = 1) to high (*n* = 5) risk of bias investigated the effect of traditional counselling (without the use of theoretical behaviour change strategies) compared with screening alone (Table 4). Interventions involved between 1 (5 min) and 20 counselling sessions and follow-up measures were taken between 3 months and 17 years from baseline. All intervention arms that involved nurse counselling following screening demonstrated significantly higher post-intervention changes in anthropometric, physiological or behaviour change outcomes, compared to screening. Significant changes were reported for: weight reduction (25,26), systolic and diastolic blood pressure reduction (26,27), cholesterol profile improvements (*n* = 3) (26–29), favourable dietary intake by self-report (26,28,30) and quantitative biomarkers (*n* = 3) (28). Significant intervention effect was not maintained at 17-year follow-up (27).

**Table 4:** PHC nurse counselling (non-behavioural) for lifestyle change compared with screening: intervention description, participant characteristics and outcomes of studies that compared effects. Results presented according to risk of bias, with lowest risk of bias first

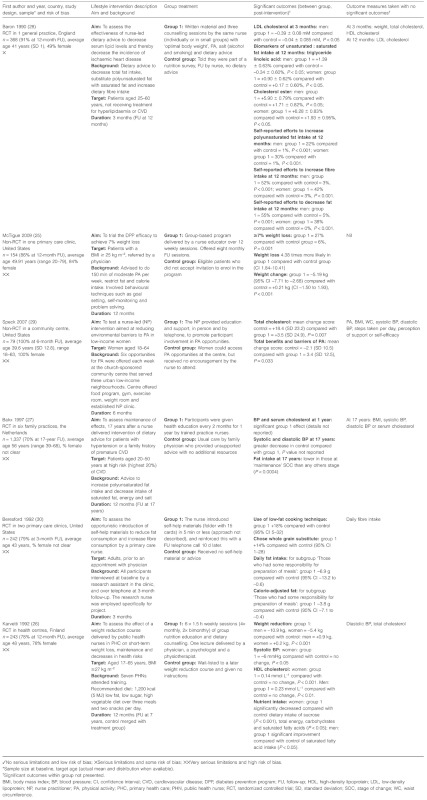

### PHC nurse lifestyle counselling based on behaviour change theories compared to traditional counselling

Four studies with a high risk of bias reported testing PHC nurse delivery of the same dose of counselling comparing traditional counselling with counselling based on behaviour change theory (31–34). Three interventions were delivered in three to five contacts, each reported significantly greater intervention effect for participants who received behavioural counselling than traditional counselling (Table 5). No intervention effect was reported when 5 min of counselling tailored to the participants' stage of change was compared to usual care or provision of written material only (34).

**Table 5:** Same dose of PHC nurse delivered lifestyle counselling based on behaviour change theories, compared to traditional counselling: intervention description, participant characteristics and outcomes of studies that compared effects. Results presented according to risk of bias, with lowest risk of bias first, then reversed chronologically

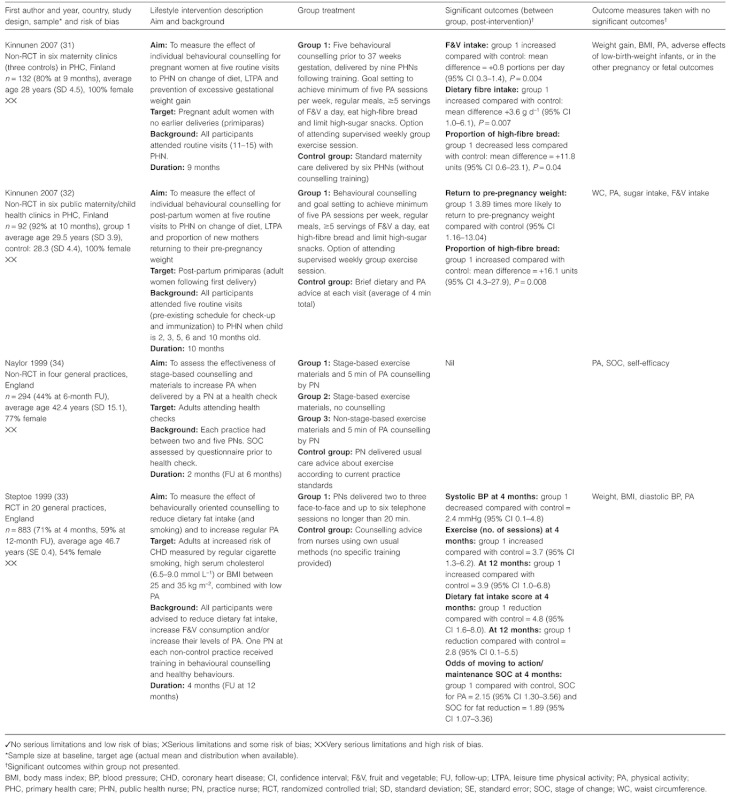

Four studies with a low (20) and high (35–37) overall risk of bias compared a low dose of traditional counselling with a higher dose of behavioural counselling (Table 6). High-quality evidence with a low risk of bias supports the use of a high dose (13 contacts) of behavioural counselling to improve patient satisfaction (20). The studies with a high risk of bias indicate that higher doses of counselling based on theories of behaviour change may result in significantly higher changes than low doses of traditional counselling, and these are evidenced by changes in: anthropometry (32,35), blood pressure (33), cholesterol profile (35), physical activity (33,37), dietary intake (31,32,35), stage of readiness and intention for behaviour change (37).

**Table 6:** PHC nurse delivering a low dose of non-behavioural counselling compared with higher dose of behavioural counselling: intervention description, participant characteristics and outcomes of studies that compared effects. Results presented according to risk of bias, with lowest risk of bias first, then reversed chronologically

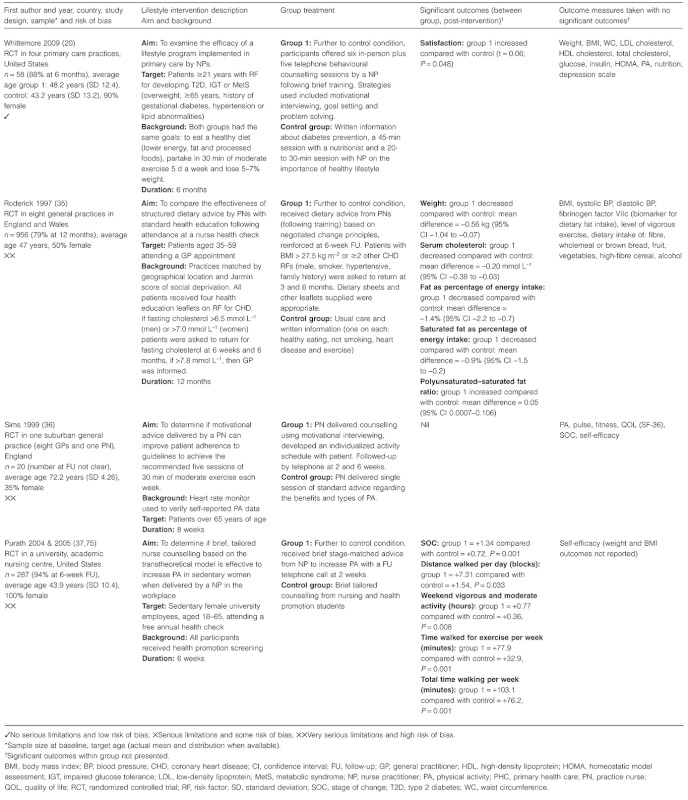

### Low dose of counselling compared to high dose

Four studies tested the effect of a low dose of counselling (one or two contacts) compared with a higher dose (≥3, Table 7) of traditional counselling (38–40) or behavioural counselling (41). Self-reported dietary intake was significantly improved when three additional brief (3–5 min) counselling sessions were delivered (38). Adherence to recommendations and self-reported physical activity were significantly higher when up to 20 additional contacts were delivered (41).

**Table 7:** Low dose of counselling compared to high dose: intervention description, participant characteristics and outcomes of studies that compared effects. Results presented according to risk of bias, with lowest risk of bias first

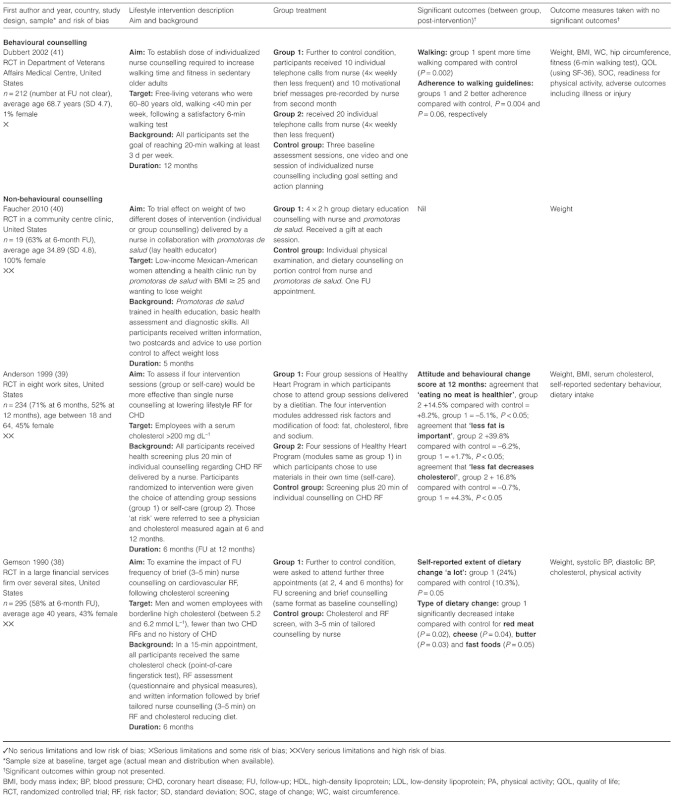

### Use of prompts or diagnostic tools

A prescription for physical activity was delivered by a nurse in one study with a low risk of bias (18) (group 1). However, the treatment effect may not be attributable to this prescription component, hence this group was excluded from further analysis.

One intervention, with a moderate risk of bias, reported that the immediate provision of cholesterol readings using point-of-care equipment did not result in significantly different cholesterol readings at 3-month follow-up (42) (Table 8). Another supports the provision of a written prompt regarding high-fibre dietary choices and reduced fat options to improve fruit and vegetable intake and reduce weight (43) (group 3). The provision of high potassium, low sodium table salt to encourage reduction of sodium chloride intake resulted in adverse side effects and was not recommended (43) (group 2). A RCT with a high risk of bias supported dietary counselling by nurses, providing some evidence that training nurses in the use of a dietary risk assessment tool resulted in significant effects on dietary intake and weight change of participants over 3 months, with changes in dietary intake maintained over 12 months (44). Another study with a high risk of bias reported that nurse delivery of a written prompt did encourage participants to seek health-related information from their general practitioner (45).

**Table 8:** PHC nurse's use of prompts or diagnostic tools: intervention description, participant characteristics and outcomes of studies that compared effects. Results presented according to risk of bias, with lowest risk of bias first

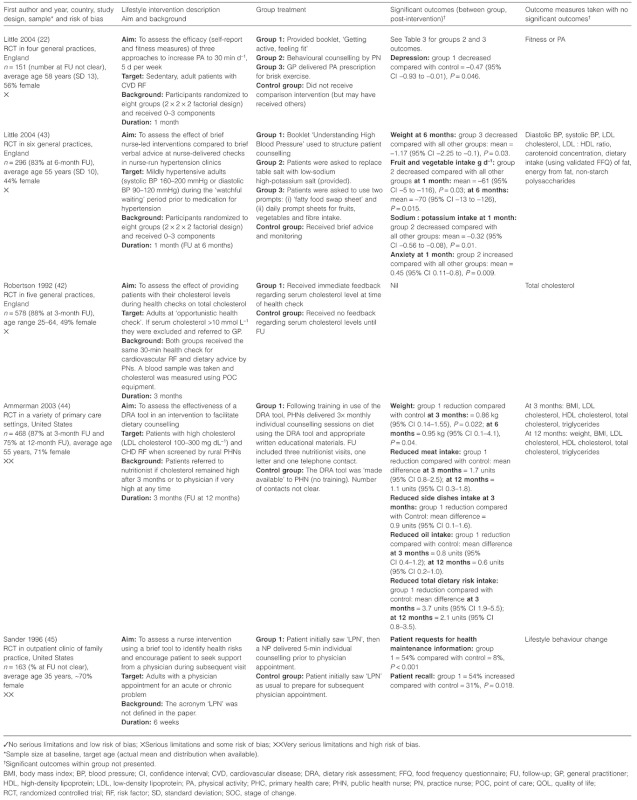

## Discussion

This is the first systematic review to synthesize the international evidence regarding the effectiveness of non-pharmaceutical lifestyle interventions for adults with the aim of reducing risk factors for preventable chronic diseases associated with obesity that were delivered by PHC nurses in a PHC setting. This synthesis contributes to the existing knowledge regarding the effectiveness of: nursing interventions in PHC to achieve changes in lifestyle risk factors for cardiovascular disease (10,46–49); lifestyle interventions to prevent cardiovascular disease (9,50,51) and manage obesity (8,52,53); lifestyle interventions in PHC (54,55); and prevention and health promotion in nursing (56).

The U.S. Preventive Services Task Force (USPSTF) concludes that changes in physiological measures such as glucose metabolism, lipid levels, blood pressure, as well as weight loss provide indirect evidence of intervention effect on long-term health outcomes (57), and these diverse measures are reflected in the interventions included in this review. The significant outcomes indicate that healthy lifestyle interventions delivered by PHC nurses can be effective over a variety of anthropometric, physiological and behavioural risk factors for chronic diseases associated with obesity.

The effectiveness of lifestyle interventions delivered by nurses, given appropriate training, is comparable to delivery by other PHC professionals with no adverse effects (17,21). This is consistent with existing literature regarding the effectiveness of nurses in PHC when compared to a PHC physician (7,9,58–62).

The USPSTF was unable to locate evidence regarding the effectiveness of screening for obesity alone (63,64). However, the provision of coronary risk information, with or without counselling, has proven effective in increasing intent to commence therapy (65). In any prospective controlled trial, the process of data collection and screening for eligibility is likely to act as an intervention in itself; hence, it is very difficult to assess the effect of an intervention compared with no intervention. Screening for risk is an essential antecedent to intervention in PHC (55), hence an essential component of lifestyle intervention to prevent chronic diseases associated with obesity. However, evidence in this review, although of mixed quality, consistently supports the provision of some dose of counselling (1–20 contacts) by nurses compared to screening alone.

The USPSTF recommends that clinicians offer high intensity counselling (≥2 contacts per month for 3 months, or a total of 6 h) and behavioural interventions to achieve weight loss in obese adults (57) and reduce cardiovascular risk factors in adults (50), or medium intensity counselling (between 31 min and 6 h) to affect significant changes in dietary and physical activity behaviours (50,55). The results of this systematic review support this, as results indicate that delivery of counselling in three or more contacts may result in significantly higher change in self-reported behaviour change for dietary and physical activity behaviours. However, there was insufficient evidence to support the use of a higher dose of intervention when assessed using anthropometric or physiological outcomes such as weight, blood pressure, cholesterol profile or fitness. There was little evidence to support low intensity counselling; however, take-home written prompts may be a useful adjunct to nurse counselling interventions in PHC.

Counselling for lifestyle change in PHC has traditionally taken the form of advice regarding recommendations to meet guidelines. More recently, behavioural counselling in lifestyle interventions has been based on psychological theoretical frameworks such as the theory of planned behaviour (66), concepts such as the transtheoretical model of health behaviour change (67), and the use of strategies such as motivational interviewing (68) and goal setting (69). Results of this review indicate that behavioural counselling strategies delivered by nurses in PHC have an effect on increasing participants' readiness for change and establishing intent for behaviour change. Those interventions that conducted a sub-analysis on participant stage of change reported that the greatest benefit was gained in the subgroup of participants that moved from an early stage of change (pre-contemplation of contemplation) to a later stage (action or maintenance). This review lends further support to the building literature describing the outcomes of counselling in PHC (50,54,70–72).

### Limitations of included articles

Many articles did not sufficiently report the methods of randomization, allocation concealment, blinding of outcome assessment, or describe conducting a power calculation to determine target sample size. This may indicate that either of these were not performed, or that they were not reported adequately. Only three of the included articles were considered to have no serious methodological limitations and a low risk of bias. It is necessary to acknowledge the difficulties involved in running RCTs with a low risk of bias in free-living populations, especially those that aim to test the effectiveness of non-pharmaceutical preventive healthcare interventions such as dietary, physical activity or other lifestyle change (73).

Small numbers of participants and high attrition rates may have limited the ability of some studies to reach significance for some outcomes and result in over-reporting of outcomes in the absence of intention to treat analyses. The included studies mainly recruited participants with high motivation to participate and few studies indicated that they used process measures to monitor the realization of intervention delivery. Short intervention duration, lack of long-term follow-up and low variety of outcome measures limited the ability of some interventions to evaluate intervention effect.

### Limitations of this systematic review

The heterogeneity of the included studies limited the opportunity for quantitative synthesis of outcome effect. The strength of recommendations is limited by the small number of studies within each comparison group analyses and the high risk of bias of the majority of studies. Authors were not contacted for extra information; hence missing information may reflect reporting bias, not necessarily limitations in the implementation rigour.

## Conclusions

The evidence supports the effectiveness of lifestyle intervention delivered by nurses in PHC to affect positive changes on a variety of outcomes associated with the prevention of chronic disease associated with obesity including weight, blood pressure, cholesterol, dietary and physical activity behaviours, patient satisfaction and quality of life. Outcomes were significantly higher if nurses provided at least one counselling session following initial screening for health risk.

This systematic review synthesizes the best available evidence in the context of informing future lifestyle interventions delivered by nurses in PHC. Further research is needed that: (i) has a low risk of bias; (ii) uses a variety of outcome measures that reflect known risk factors for chronic disease including anthropometric, physiological, behavioural and psychosocial intervention effects; (iii) explores training requirements for effective nurse delivery of lifestyle interventions; (iv) explores the efficacy of counselling using theoretical frameworks for behaviour change; and (v) examines the effect of dose.

## Supporting Information

**Table S1.** MEDLINE (OVID) search strategy conducted 13-9-10, highlighting the six groupings of search terms.

**Table S2.** Risk of bias of included articles (Supporting Information for Table S1). Each category scored as Adequate (A), Inadequate (I), Not done (N) or Unclear (U).
