# Supplementary material for: Nurse delivered lifestyle interventions in primary health care to treat chronic disease risk factors associated with obesity: a systematic review
Source: Obes Rev. 2012 Dec;13(12):1148–71. doi: 10.1111/j.1467-789X.2012.01029.x (PMC3533768; doi:10.1111/j.1467-789X.2012.01029.x)
Supplement: Supplementary file 1 [file obr0013-1148-SD1.doc]

**Table S1**: Medline (OVID) search strategy conducted 13-9-10, highlighting the six groupings of search terms.

| 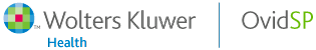 | | Logged in as Ginny Sargent at ANU | | --- | | [My Account](http://ovidsp.tx.ovid.com/sp-3.2.1/ovidweb.cgi?&S=OGDBFPMJNNDDDIAONCDLDDGCODGFAA00&Access+Personal+Account=1)[Help](http://www.ovid.com/site/help/documentation/ospb/en/advanced.htm)[Logoff](http://ovidsp.tx.ovid.com/sp-3.2.1/ovidweb.cgi?Logoff=1&S=OGDBFPMJNNDDDIAONCDLDDGCODGFAA00) | |
| --- | --- | --- | --- |

1 exp Health Services/ 1285279

2 exp comprehensive health care/ or exp primary health care/ 171294

3 primary health care.mp. 48367

4 (primary care or ambulatory care).mp. 94674

5 (family practi$ or general practi$).mp. 93834

6 (health centre$ or health center$ or health service$).mp. 289688

7 (community care or community health centre$).mp. 3110

8 ((nurse or nurse-led) adj clinic$).mp. 7326

9 1 or 2 or 3 or 4 or 5 or 6 or 7 or 8 1538085

10 exp faculty, nursing/ or exp health educators/ or exp nurses/ or exp nursing staff/ 111830

11 (nurs$ or health educator$).mp. 488572

12 (nurs$ adj home$).mp. 31196

13 11 not 12 457376

14 10 or 13 459648

15 (evaluat$ or effect$ or evidence or compar$).ti. 2006999

16 (assess$ or impact or impact assess$).ti. 254026

17 (manag$ or treat$ or therap$ or deliver$).ti. 1575472

18 (intervention$ or investigat$ or result$ or study or trial$ or program$).ti. 1267290

19 15 or 16 or 17 or 18 4570264

20 exp preventive health services/ or secondary prevention/ 354813

21 ((behaviour or behavior) and (change or modification)).mp. 54478

22 exp health behavior/ or exp motivation/ or exp behavior control/ or exp behavioral medicine/ or exp behavioral research/ 187708

23 exp Exercise/ or (physical activit$ or sedentary behavio?r$).mp. 78244

24 exp feeding behavior/ or exp nutrition disorders/ or exp overnutrition/ or exp obesity/ or exp Food Habits/ 271798

25 chronic disease.mp. or Chronic Disease/ 203233

26 lifestyle.mp. or exp Life Style/ or exp Health Promotion/ or exp Health Education/ 213293

27 20 or 21 or 22 or 23 or 24 or 25 or 26 1108850

28 exp body weight/ or exp overweight/ or exp obesity/ 287308

29 exp Overnutrition/ 103169

30 exp body weight changes/ or exp weight gain/ or exp weight loss/ 37622

31 (overweight or obese or obesity or weight or skinfold$ or anthropom$ or BMI or body mass index or waist circumference).mp. 860478

32 (diet$ or healthy eating or physical activity).mp. 445377

33 28 or 29 or 30 or 31 or 32 1193427

34 9 and 14 and 19 and 27 and 33 1563

35 (prader willi or prader-willi).ti. 1500

36 (schizoph$ or psychiatric patient$ or lamotrigine).ti. 56974

37 (child$ or pediatric$ or paediatric$ or infant$ or $pubert$).mp. 2038870

38 (fall$ or cancer$ or chemotherapy$ or hospital$ or qualitative).ti. 731087

39 (nurs$ adj home$).ti. 10709

40 35 or 36 or 37 or 38 or 39 2774052

41 34 not 40 1049

42 limit 41 to english language 970
