# Supplementary material for: Nurse delivered lifestyle interventions in primary health care to treat chronic disease risk factors associated with obesity: a systematic review
Source: Obes Rev. 2012 Dec;13(12):1148–71. doi: 10.1111/j.1467-789X.2012.01029.x (PMC3533768; doi:10.1111/j.1467-789X.2012.01029.x)
Supplement: Supplementary file 2 [file obr0013-1148-SD2.doc]

**Table S2**: Risk of bias of included articles (supplementary information for Table 1). Each category scored as Adequate (A), Inadequate (I), Not done (N) or Unclear (U).

| First author and year | Level/Method of random sequence generation | Random sequence generation-adequacy | Method of allocation concealment | Allocation concealment-adequacy | Recipient & method of blinding | Description of power/sample calculation | Effective sample size reached | Groups similar at baseline | Description of inclusion /exclusion criteria-adequacy | Intent-to-treat analysis performed | Outline diffs between groups-baseline or PI | Paper discussed differences between completers and non-completers | Outcome data for >80% | Treatment of missing data | Analysis method | Evidence of outcome measure reporting bias |
| --- | --- | --- | --- | --- | --- | --- | --- | --- | --- | --- | --- | --- | --- | --- | --- | --- |
| Balch 1976 ^21^ | 'Subjects were randomly assigned to one of four groups' (no detail of method) | U | Not described | U | No blinding described | Nil | No | Yes | A | No | 'No significant differences amongst groups' at baseline. Participants in Group 3 (social worker-led) had higher average % overweight | No | No | Excluded from analysis | Not clear |  |
| Baron 1990 ^25^ | 507 potential participants "randomly chosen from over 20 000 patients on the lists", 437 eligible patients then "randomly assigned" to group | U | Allocation prior to invitation | N | No blinding described, control participants told they were involved in a nutrition survey, contamination likely as all participants in same practice, and both groups delivered by same nurse | Nil | No | No | A | No | Signif lower smoking in Grp 1 men (32 vs 48%), higher % of women in social class 1 or 2 in Grp 1 (43 vs 24%). Those that reported their family did not like the recommendations were more likely to not complete | Yes | Yes | None given, only analysed those that completed assessment | Comparison of means, appropriate analysis (could have compared difference in change) | Plasma glucose results not presented. No measure of incidence of ischaemic heart disease (aim) |
| Gemson 1990 ^38^ | 'assigned randomly to one of two groups' | U | Not described | U | No blinding described | Nil | No | Yes | A | No | No significant differences between groups or non-completers | Yes | No | None given, only analysed those that completed follow-up | Not clear | Demographics, and physical variables not reported |
| Beresford 1992 ^27^ | Participant allocation according to presence of point-of-care cholesterol monitor in the practice. Time-slots were not random. Inconsistencies in allocation resulted in exclusion of results from 2 practices and other patients | I | according to presence of point-of-care cholesterol monitor | I | No blinding described | That 8 practices would be necessary to detect a between group difference of 0.15mmol/L (p,0.05, power 0.90) | No | No | I | No | Women Grp 1 had significantly higher (p<0.05) mean cholesterol at baseline (5.58 v 5.35mmol/L) | No | Yes | Patients not returning for follow-up were assumed to have made no change to behaviour or cholesterol level from baseline | Appropriate. Men and women analysed separately to account for between group differences in proportion of females | BP, height, weight and dietary data taken as part of health check not reported |
| Karvetti 1992 ^23^ | Sample "selected for the study were randomly assigned to a treatment and a control group" | U | Not described | U | No blinding described | Nil | No | No | A | No | Significant of baseline differences not clearly presented, differences apparent in female weight, SBP & DBP. Intervention did calculate significance of change, not absolute, so will have allowed for this. StateNo signif baseline diffs for nutrient intake. | No | No | Only analysed data from those that completed (and complied) with study | Appropriate | r |
| Robertson 1992 ^42^ | Randomisation by clinic days, balanced in 10 day blocks. | I | By day of presentation at clinic. Intervention days may have been predictable | I | Nurse did not meet with control group. Data collected by an RA (in person or over telephone) who was not privvy to allocation (but would have known if nurse not present at clinic at time of baseline interview) |  | No | Yes | A | No | Completers less likely to be African American, and more likely to have >12th grade education | Yes | No |  | Appropriate |  |
| Neil 1995 ^17^ | Randomisation was done using a list of consecutive random treatment assignments. Patients living at the same address were randomised to the same intervention group to avoid contamination | A | The study coordinator randomised the eligible patients to one of the three interventions before they next attended | A | Laboratory blind to allocation. The results of lipid and lipoprotein measurements were not entered in the clinical case notes until the end of the trial to avoid the possible confounding effect of dietary advice offered opportunistically | calculated SS not reported. "The trial was designed to have a 90% statistical power to detect a difference of >/=0.3 mmol/l between the groups in mean total cholesterol concentration at the end of the trial with a 5% level of significance" | No. Not reported | No | A | Yes | The mean total cholesterol concentration and percentage of smokers were significantly higher in the control group compared with Grp 2 and both other groups respectively | No | Yes | Intention to treat with no substitution of missing data | Appropriate |  |
| Sander 1996 ^45^ | 'randomized by the day they presented' | I | None | I | Physician was unaware of which patients were in the study (presumably presence of the card would indicate this) |  | No | No | A | No | "Generally surprising uniformity in the demographics across groups" | No | No | Not reported, presumed excluded from analysis | Not clear |  |
| Bakx 1997 ^24^ | Not described | U | Not described | U | No blinding described |  | No | No | I | No | Systolic and diastolic BP higher at baseline in control group | No | No | Not described, but appear to be excluded only reported outcomes for n=472 not 938 | Not clear |  |
| Roderick 1997 ^35^ | Matched pairs of general practices, one allocated to each group | U | Not described, cluster randomisation may assist | U | No blinding described, cluster randomisation may assist | Aimed to recruit 1200, for 80% power on an ITT analysis of 8% difference in serum cholesterol at p=0.05 | No | No | A | Yes | Groups different in % smokers and % in manual labour (both higher in control group). Significance not discussed. Completers more likely to be in non-manual labour & non smokers. | Yes | No | Intention to treat, baseline measures used if follow-up data missing | Appropriate. Changes presented in changes in means for each practice | Pulse outcomes not reported |
| Anderson 1999 ^39^ | Quasi-experimental design with cluster randomisation, "eight worksites were randomly assigned" to either control or intervention group | U | Not described | U | None |  | No | No | I | No | Significant baseline differences between group in grams of fat eaten/day (control: 37.8, Grp 1: 45.1, Grp 2: 57.3), serves of meat eaten/day (0.83: 1.34: 1.14), and attitude regarding importance of eating less fat (57.8: 80.6: 51.7) | No | No | None | Not clear |  |
| Naylor 1999 ^34^ | None | N | None, done according to preference and skills | N | PNs were blinded to the "true nature of the intervention" |  | No | No | I | No | Baseline level of PA similar, but significantly higher self-efficacy, younger age and more females in Grps 1 & 3. Non-completion was not associated with baseline SOC | Yes | No | Not described, presume excluded | Appropriate |  |
| Sims 1999 ^36^ | Not described | U | Not described | U | None described | None | No | No | I | No | The mean pulse in Grp 1 & hence % time with BP>100 a lot higher at baseline than control (p=0.06). Not significant but close considering such a small sample. This definitely skews results throughout | No | No | None described | Appropriate |  |
| Steptoe 1999 ^33^ | 20 practices were allocated using minimisation for three factors: Jarman SES score, PN practice hours, fundholding status (an alternative to stratification that balances multiple factors and likely to produce comparable groups) | A | Prediction of allocation based on factors unlikely in advance | A | Practices were assigned by a statistician who was blinded to the practice identity. Practices were informed of their status (not blinded) | Target 100 patients per practice. Taking intracluster correlations of risk factors into account, this was calculated to detect a decrease of 0.27 mmol/l in total serum cholesterol concentration with 90% power at the 5% significance level | No | Yes | A | No | Patients lost to FU were younger than completers, no diff on sex, education, occupation, family history of CVD or readiness for change | Yes | No | None given, only analysed those that completed assessment | All between group comparisons, but no evidence that significance of difference was calculated. Statistical comparison of intervention and control groups was carried out with weighted means for each practice thereby taking account of cluster effects | Self-efficacy outcomes not reported |
| Gold 2000 ^30^ |  | N | None | N |  |  | No | No | I | No | Control group had significantly higher risk score at baseline (6.24 v 5.9, p<0.01). Control grp was slightly older (mean age 46 v 45) and had more women (45% v 42%), significance not described | No | No | None given | Appropriate |  |
| Dubbert 2002 ^41^ | Three group RCT, randomised following baseline (possibly allocated at first counselling-method of allocation not described) | U | " The nurse explained the folllow-up assignment to participants as they completed their counseling" | U | Baseline data collected prior to allocation, and F-U data collector blinded to group assignment |  | No | Yes | A | Yes | Initiation (first months adherance to guidelines) considerably higher in Grp 1, even though treatment exactly same as Grp 2 until second month | No | Yes | None, "modified" intention to treat ie excluded those who withdrew or did not attend first session | Appropriate |  |
| Ammerman 2003 ^44^ | Randomization was by county (health department), stratified by region (east/west) to account for differences in demographics | U | Not described | U | No blinding described, U if health departments aware of allocation | Target 180 participants in each group (30 participants in each county), alpha 0.05, power 80% | Yes | No | A | No | PHN in Grp 1 had more years of nursing experience. Participants in the control group had significantly higher level of CHD risk factors and significantly higher % with history of hypertension. Males in the control group had lower levels of HDL-C and LDL-C | No | Yes | Reported only those for which outcome data was recorded, although reported that when missing data reported as zero change for dietary assessment 'results were similar' | Appropriate, accounted for baseline differences | Self-efficacy, stage-of-change and readiness outcomes not reported |
| Aittasalo 2004 ^29^ | Volunteers were randomised separately for each company | U | Study group "was not revealed to the subjects until the baseline information about LTPA was collected" | U | No blinding described |  | No | No | A | No | Some differences (around education and SES), significance not described, but allowed for in results | No | Yes | NA | Appropriate |  |
| Little 2004 ^28^ | Patients were randomly assigned to one of eight groups, in a balanced 2 Χ 2 Χ 2 factorial design, by opening a sealed opaque numbered envelope that had been prepared previously at the trial centre by the research nurse (probably random number tables) | A | Instruction sheets according to group allocation were sealed in opaque, numbered envelopes at the study centre and distributed to each practice in opaque envelopes. | A | Assessment carried out by research assistant, not privy to allocation group | Calculated SS of 128, alpha 0.05, beta 0.2 for 0.5SD change in all main outcomes | Yes | No | A | Yes | Higher intention to exercise in Grp 3 | No | Yes | Intention to treat, baseline data substituted for missing outcome data | Appropriate, but no treatment control group absent due to factorial design | Authors discounted weight and cholesterol findings |
| Little 2004 ^43^ | Randomisation using random number tables took place several weeks in advance at the study centre | A | Instruction sheets according to group allocation were sealed in opaque, numbered envelopes at the study centre and distributed to each practice | A | The same nurses delivered all groups according to allocation-potential for contamination | Calculated 198, target 240 patients to allow for 20% attrition, alpha=0.05, beta=0.2, to detect change of 3mm Hg | Yes | Yes | A | Yes | 'well balanced' at baseline, F&V consumption reportedly 'slightly higher than expected' | No | Yes | Intention to treat with no substitution of missing data | Appropriate |  |
| Purath 2004 & 2005 ^37, 75^ | Randomisation by university building prior to recruitment | U | Prior to recruitment, but colleagues of those already screened then aware of intervention group | I | No blinding described |  | No | Yes | A | No | No significant differences at baseline. Non-competers were significantly lower educated | No | Yes | Excluded | Appropriate | Weight and BMI outcomes not reported |
| Kinnunen 2007 ^32^ | None | N | None | N |  | Target 40 participant in each group (from 6 clinics) | Yes | Yes | A | No | Participants who dropped out of the study (n = 7) were younger, less educated and had higher pre-pregnancy and postpartum BMI, but lower gestational weight gain and weight retention at 2 months postpartum | Yes | Yes | None given, only analysed those that completed assessment | ANCOVA for repeated measures, adjusted for confounders |  |
| Kinnunen 2007 ^31^ | None | N | None | N |  | Target 40 participant in each group (from 6 clinics) | Yes | No | A | No | Women in the intervention group were younger, less educated, more often smokers and they had higher pre-pregnancy weight and BMI on average than the women in the control group | No | Yes | None given, only analysed those that completed assessment | ANCOVA for repeated measures |  |
| Speck 2007 ^26^ |  | N |  | N |  |  | No | Yes | A | No |  | No | Yes | Not described | Appropriate | LDL cholesterol, triglycerides and glucose outcomes not reported |
| Lawton 2009 & Rose 2007 ^18, 19^ | Computer generated, random sequence by independent researcher | A | Allocation concealed until following baseline assessment | A | Assessors at baseline, 12M and 24 M were blind to group allocation | 7% difference in proportion of women reaching target PA, alpha=0.05, 80% power, allowing for 10% attrition | Yes | Yes | A | Yes |  | No | Yes | Baseline data used for missing data at FU | Appropriate | Fitness outcomes not reported |
| McTigue 2009 ^22^ | None | N | None | N | None |  | No | No | A | No | intervention group (self-selected) were slightly older (53 v 47 years, p<0.004) | No | Yes | Reported baseline and analysed outcomes only for those that with outcome data complete | Appropriate | BMI outcomes not reported |
| Whittemore 2009 ^20^ | Practices randomised using a computerised table of random numbers | A | Practices not likely to be aware prior to allocation | A | Data collection by research and lab personnel blind to allocation. Nutritionist blind to allocation | 20% of what would be necessary for an RCT | No | No | A | Yes | At baseline, Grp 1 older, higher SES, diff ethnicity (all signif?). Non-completers younger, higher BMI, lower LDL (all p<0.05) | Yes | Yes | Missing data substituted by mean | Appropriate BUT stats on Table 2 are inconsistent with text. |  |
| Faucher 2010 ^40^ | "Women were randomised to one of two groups" | U | "Women were randomised to one of two groups". No allocation concealment described | I | No blinding described | With a mean weight loss of 10 lb and an SD of 5, 80% power and p=0.05, need 20 per group | No | Yes | A | No |  | No | No | None | Appropriate |  |

✓ no serious limitations and low risk of bias

🗶 serious limitations and moderate risk of bias

🗶🗶 very serious limitations and high risk of bias
